# Evaluation of energy and dietary intake estimates from a food frequency questionnaire using independent energy expenditure measurement and weighed food records

**DOI:** 10.1186/1475-2891-9-37

**Published:** 2010-09-15

**Authors:** Monica H Carlsen, Inger TL Lillegaard, Anette Karlsen, Rune Blomhoff, Christian A Drevon, Lene F Andersen

**Affiliations:** 1 Department of Nutrition, Institute for Basic Medical Sciences, University of Oslo, Oslo, Norway

## Abstract

**Background:**

We have developed a food frequency questionnaire (FFQ) for the assessment of habitual diet, with special focus on the intake of fruit, vegetables and other antioxidant-rich foods and beverages. The aim of the present study was to evaluate the relative validity of the intakes of energy, food and nutrients from the FFQ.

**Methods:**

Energy intake was evaluated against independent measures of energy expenditure using the ActiReg^® ^system (motion detection), whereas 7-days weighed food records were used to study the relative validity of food and nutrient intake. The relationship between methods was investigated using correlation analyses and cross-classification of participants. The visual agreement between the methods was evaluated using Bland-Altman plots.

**Results:**

We observed that the FFQ underestimated the energy intake by approximately 11% compared to the energy expenditure measured by the ActiReg^®^. The correlation coefficient between energy intake and energy expenditure was 0.54 and 32% of the participants were defined as under-reporters. Compared to the weighed food records the percentages of energy from fat and added sugar from the FFQ were underestimated, whereas the percentage of energy from total carbohydrates and protein were slightly overestimated. The intake of foods rich in antioxidants did not vary significantly between the FFQ and weighed food records, with the exceptions of berries, coffee, tea and vegetables which were overestimated. Spearman's Rank Order Correlations between FFQ and weighed food records were 0.41 for berries, 0.58 for chocolate, 0.78 for coffee, 0.61 for fruit, 0.57 for fruit and berry juices, 0.40 for nuts, 0.74 for tea, 0.38 for vegetables and 0.70 for the intake of wine.

**Conclusions:**

Our new FFQ provides a good estimate of the average energy intake and it obtains valid data on average intake of most antioxidant-rich foods and beverages. Our study also showed that the FFQs ability to rank participants according to intake of total antioxidants and most of the antioxidant-rich foods was good.

## Background

Assessment of long-term dietary intake using methods of self-reporting has generally been associated with measurement errors [1]. Methods used challenges the participants' memory and ability to take into account the variability in intake, from day to day or by season. Because of the measurement errors, dietary assessment methods should always be validated before use. We have developed a new food frequency questionnaire (FFQ) for the assessment of habitual diet among adult Norwegians. In nutrition research FFQs are extensively used to investigate relationships between food intake and health [1]. Although the FFQ has inherent measurement errors, the FFQ designed to measure a person's habitual dietary intake over a defined period of time, is relatively inexpensive and easy to administer and is the exposure assessment tool of choice for large nutritional epidemiologic studies [1,2]. Our present semi-quantitative FFQ was developed for the assessment of habitual diet with special focus on the consumption of fruit, vegetables and other antioxidant-rich foods and beverages.

Assessment of energy intake (EI) is crucial in any diet assessment, because all other nutrients are provided within the quantity of food needed to fulfill the energy requirement. Intake estimates of many foods and nutrients will be inaccurate if the EI is inaccurate. Thus, it is important to establish the FFQ's ability to assess EI when a new questionnaire is developed. The aims of the present study were to i) evaluate the EI from our new FFQ with an independent energy expenditure (EE) measurement; and ii) to assess the relative validity of the intakes of fruits, vegetables and other antioxidant-rich foods from the FFQ, using a 7-days weighed food record (WR) as reference method.

## Methods

### Subjects and study design

The evaluation study was designed to include a representative sample of the Norwegian population. The participants were recruited via mail to 4500 randomly selected women and men with home addresses in the Norwegian capital or surrounding area. The random selection of subjects was administered by The National Tax Office/Population Registration Office, and an equal number of men and women were invited, aged 18 to 80 years. Five hundred and four invited men and women (11%) responded to the invitation. After interview screening 346 participants were enrolled in the study, of which 232 took part in the present part of the evaluation study. Exclusion criteria were pregnancy, weight loss of more than 5 kg during the last 6 months prior to the study and participation in other research projects. Data collection was carried out from September 2006 until October 2007. After recruitment the participants received the FFQ and written instructions by mail and were asked to fill in the FFQ at home. Within two weeks the participants attended a physical examination and returned the FFQ. Measurements of height and weight were performed by trained staff and the participants were lightly dressed with indoor clothes and without shoes when the anthropometric measures were taken. At the physical examination, the participants were randomly assigned to participate in either the assessment of EE (n = 64), or to conduct the 7-days WR (n = 168). The participants were given both oral and written instructions how to perform the tasks. The EE measurements and the 7-days WR were initiated 3 to 4 weeks after the participants filled in the FFQ. Social economic status of the participants was not assessed.

This study was conducted according to the guidelines laid down in the Declaration of Helsinki and all procedures involving human subjects were approved by the Regional Ethics Committee for Medical Research. Written informed consent was obtained from all subjects.

### The semi-quantitative food-frequency questionnaire

The 14-page questionnaire was designed to capture the habitual food intake among Norwegian adults the preceding year. The FFQ was an extended and revised version of the semi-quantitative food-frequency questionnaire used in the Norwegian nation wide survey NORKOST 1997 (NFFQ). The original NFFQ was a validated, 180 items optical readable FFQ, developed to cover 100% of the total EI of the population [3-6]. Based on our extensive screening of antioxidant content in foods and beverages [7], the NFFQ was updated and revised with questions about food products and food categories assumed to be important sources of antioxidant intake in Norway. The new FFQ included 270 food items, grouped together according to the Norwegian meal pattern. Additional questions were added concerning intake of several food categories. In detail, 19 questions about berries, 4 questions about fruit, 6 questions about vegetables, 2 questions about chocolate, 3 questions about coffee and 2 questions about tea were added. Questions concerning the variable intake of berries due to seasonal variations were included. Furthermore, 10 questions were added about nuts and seeds and 27 questions about spices and herbs. The options of frequency of consumption of particular food items varied from several times a day to never/seldom, with portion sizes based on typical household units: slices, glasses, cups, pieces, spoons and teaspoons. When frequency was answered but not portion size, the food item was given the smallest portion size. When portion size was answered but not frequency, the food item was given the value zero. One participant was excluded from the study because a substantial amount of the questions in the FFQ were left unanswered. The dietary questions totaled 11 pages of the questionnaire whereas the last 3 pages were dedicated to questions concerning dietary supplements, smoking, physical activity and past and present illnesses and medication. The answered FFQs were scanned and the image files translated into data files using the Cardiff Teleform 2006 software.

### The energy study

Sixty-four participants were randomly selected to carry out the EE measurements. The position-and-movements monitor ActiReg^® ^(PreMed AS, Oslo, Norway) was used to measure EE for 7 consecutive days. Two participants were excluded from the data analyses due to failure in the ActiReg^® ^recording system and three were excluded due to measurement periods of less than 7 days. Thus, EE data from 59 participants were available for comparison with the EI estimated from the FFQ.

The ActiReg^® ^system uses a combined second-to-second recording of body position and motion to calculate EE [8]. The monitor has two pairs of position-and-motion sensors connected by cables to a battery-operated storage unit fixed to a waist belt. Each pair of sensors is attached by medical tape to the chest and the front of the right thigh, respectively. When the participants were sleeping, the ActiReg^® ^equipment was taken off and placed in a horizontal position. Collected data were transferred to a computer and processed by a dedicated program called ActiCalc^® ^[8]. The ActiReg^® ^system has been validated against both the doubly labeled water method and whole-body indirect calorimetry among young adults [8]. The validation studies demonstrated that there was no significant mean difference between EE measured by the ActiReg^® ^system and EE measured with indirect calorimetry or the doubly labeled water method [8]. The correlation coefficients between EE measured with the ActiReg^® ^system and EE measured with indirect calorimetry or the doubly label water method were 0.86 and 0.76, respectively [9].

### The 7-days weighed food records study

One hundred and sixty-eight participants were randomly selected to do the 7-days WR. Data from 21 participants were excluded from the analyses because less than 7 days had been recorded. The participants got written and oral instructions how to weigh and record all foods and beverages consumed in a food diary for 7 days, divided into 2 periods of 4 and 3 consecutive days, one week apart, including all days of a week. Each participant was provided with a food diary and a digital scale (Phillips HR 2393/95). The WRs were manually transcribed into data files which were imported into the KBS system. The WRs were distributed to the participants over a period of 7 months, from September to March, omitting the Christmas holidays.

### Food, nutrient and antioxidant databases

Average daily intake of energy, nutrients and antioxidants from the FFQ and the WR were computed using the food database KBS AE-07 and KBS software system (KBS, version 4.9, 2008) developed at the Department of Nutrition, University of Oslo, Norway. The food database KBS AE-07 is based on the 2006 edition of the Norwegian food composition table http://www.norwegianfoodcomp.no. The antioxidant values in KBS AE-07 are based on our extensive analyses of antioxidants in more than 3100 food samples procured worldwide [7,10,11]. The total antioxidant content in foods were measured using the FRAP (ferric-reducing ability of plasma) method and is expressed as mmol/100 g sample [7,10,11].

### Statistical methods

Sample size calculation for the EI study was based on a SD of EI of 2 MJ and a significance level of 0.05 with 80 percent power [12,13]. Thirty-two participants were required to detect a mean difference of 1 MJ between EE measured with ActiReg^® ^and EI assessed with the FFQ. Height, weight, EE, EI, EI-EE and EI/EE were normally distributed and are presented as means and 95% confidence intervals (CI). Differences between EI and EE for all participants and between the genders were analyzed using paired and unpaired T-tests, respectively. All significance levels were two-sided. The visual agreement between the methods was analyzed as described by Bland and Altman [14,15] using a plot of the differences between the two methods versus the average of the measurements. This type of plot shows the magnitude of disagreement, spot outliers and any trend. The relationship between EI and EE was investigated using Pearson product-moment correlation coefficient and by cross-classification of the participants into tertiles of EE and EI. The accuracy of the reported EI was calculated by expressing the ratio EI/EE, for which a value of 1 would mean complete agreement between EI and EE. Acceptable reporters (AR) were defined as having the ratio EI/EE in the range of 0.80 to 1.20, under-reporters (UR) as EI/EE < 0.80 and over-reporters (OR) as EI/EE > 1.20. These definitions are partly based on the 95% confidence limits of agreement between EI and EE measured by the double labeled water method [16].

Chi-square tests for independence were conducted to assess if there was any difference in the distribution of men, women, smokers and non-smokers in UR and AR.

Percent energy from protein, fat and carbohydrates were normally distributed and are presented as means and SD. All micronutrient intakes and most food intake estimates were not normally distributed and data are therefore presented as median values with 25 and 75 percentiles. The differences between food intakes estimated by the FFQ and the WR were tested using Wilcoxon signed rank test (paired data). The relationship between the methods was analyzed using the Spearman rank correlation coefficient or the Pearson product-moment correlation coefficient and by classifying participants into quartiles of intake. Correctly classified participants were defined as participants categorized in the same quartile as defined by the WR estimate, whereas 'grossly misclassified' participants were defined as participants classified into opposite quartile. The accuracy of the estimated intakes was explored by expressing the ratio of estimates (FFQ/WR) for all nutrients and foods. Energy adjustment of intakes was calculated as absolute intake per 10 MJ. Results were considered to be statistically significant at p < 0.05. Data were analyzed using SPSS for Windows release 16.0 (SPSS Inc., Chicago, IL, USA).

## Results

### Energy intake and energy expenditure estimates

**Figure 1:**
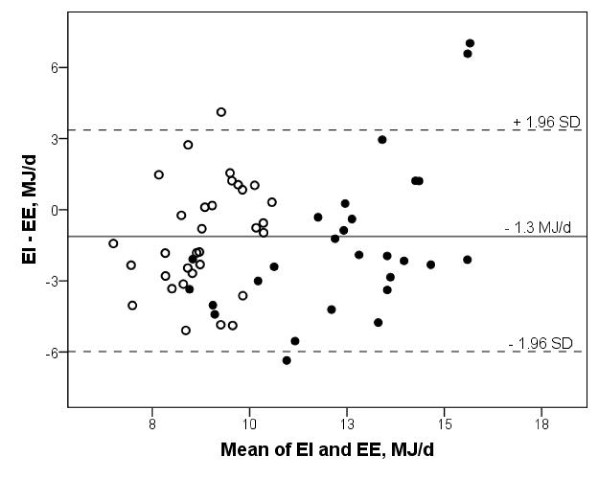
**Energy intake and expenditure in men and women**. Bland-Altman plot of the difference between energy intake (EI) and energy expenditure (EE) against the mean of EI and EE for each participant, men (●) and women (○). The differences in EI - EE are normally distributed. The solid line represents the average difference between EI and EE. The dotted lines are upper and lower limits of agreement (mean ± 1.96 SD) in this population.

The 59 participants had a mean age of 44 (95% CI 40, 48) years. The mean EI from the FFQ was significantly lower than the mean EE (p < 0.01) (Table 1) and underestimated by 10.6% on average. The same difference between methods was observed for both men and women separately. The absolute mean difference between EI and EE was -1.3 (95% CI, -2.0 to -0.6) MJ/day with 95% confidence limits of agreement (± 1.96 SD) of -6.0 to 3.4 MJ/day (Figure 1). The difference and the ratio of the two methods were of similar magnitude and did not differ significantly between the sexes (Table 1). The Pearson correlation coefficient between the two methods was 0.54. Cross-classification of all participants into tertiles with regard to EI and EE showed that 30 of the 59 participants (51%) were classified in the correct tertile, whereas 26 participants (44%) were classified in adjacent tertiles and three participants were misclassified by two tertiles.

**Table 1:** Energy intake, energy expenditure and characteristics of participants in the energy study

	All participants, n = 59		Men, n = 27		Women, n = 32		UR, n = 19		AR, n = 35		OR, n = 5	
	Mean	CI	Mean	CI	Mean	CI	Mean	CI	Mean	CI	Mean	CI
BMI (kg/m2)	25.1	24.2, 26.1	26.3	25.1, 27.5	24.1	22.7, 25.5	26.1	24.0, 28.2	25.1	24.0, 26.1	21.7	18.2, 25.2
Current smokers (n)	16		9		7		5		11		0	
EE (MJ/day)	11.1	10.5, 11.8	13.2	12.5, 13.9	9.4	9.0, 9.9	11.3	10.3, 12.2	11.2	10.3, 12.1	10.1	6.7, 13.5
EI (MJ/day)	9.8^a^	9.0, 10.6	11.7^b^	10.4, 13.0	8.3^c^	7.6, 8.9	7.3^d^	6.6, 8.0	10.5	9.7, 11.3	14.8	9.5, 20.1
EI-EE (MJ/day)	-1.3	-2.0, -0.6	-1.5	-2.8, -0.22	-1.2	-2.0, -0.34	-3.9^d^	-4.5, -3.4	-0.7	-1.2, -0.3	4.7	2.2, 7.2
EI/EE	0.89	0.83, 0.96	0.89	0.79, 0.99	0.90	0.81, 0.99	0.65^d^	0.62, 0.68	0.94	0.90, 0.99	1.46	1.29, 1.64

Energy intake, EI; energy expenditure, EE; under-reporters, UR; acceptable reporters, AR; over-reporters, OR. Data are presented as mean values with 95% confidence intervals (CI). ^a ^Different from EE, p < 0.001, (CI: 0.6; 2.0); ^b ^different from EE, p = 0.02, (CI: 0.3; 2.0); ^c ^different from EE, p = 0.007 (CI: 0.2; 2.8). ^d ^Mean value different from AR mean value, p < 0.01.

### Energy intakes among under-reporters and acceptable reporters

**Figure 2:**
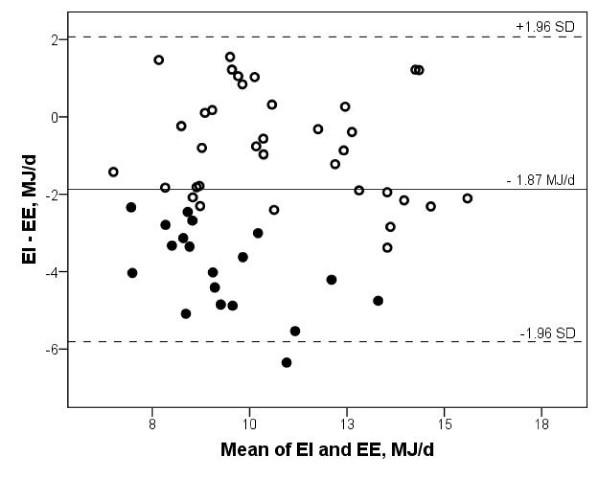
**Energy intake and expenditure in under-reporters and acceptable reporters**. Bland-Altman plot of the difference in energy intake (EI) and energy expenditure (EE) against the mean of EI and EE, under-reporters (●) and acceptable reporters (○). The solid line represents the average difference between EI and EE. The dotted lines are upper and lower limits of agreement (mean ± 1.96 SD) in this population.

Nineteen participants (32%) were classified as UR (EI/EE < 0.80) and 35 (59%) were classified as AR (0.80 < EI/EE < 1.20) (Table 1). There were no significant differences in the distribution of men and women or smokers/nonsmokers in UR and AR (p = 0.78 and p = 0.13, respectively). Moreover, BMI and age did not differ significantly between the groups (p = 0.69 and p = 0.84, respectively). EE was not significantly different (p = 0.97) between UR and AR, whereas EI, absolute mean difference and the ratio of the methods were significantly different between UR and AR (all comparisons p < 0.01, Table 1). UR underestimated their EI by on average -35% compared to EE; the AR underreported their EI by on average -6%. The Pearson correlation coefficients between EI and EE were r = 0.85 for UR as well as AR separately. The Bland-Altman plot of EI and EE for the UR and AR separately show a tendency of increasing under-reporting with increasing mean of EI and EE among the UR (Figure 2). The range defining the AR is relatively broad and if we use a more narrow range of 0.90 to 1.10 for EI/EE ratio, 17 participants would have been classified as AR.

In our study five participants were classified as OR (EI/EE > 1.20). OR overestimated their EI on average by 46%, whereas BMI and EE did not differ significantly from AR. Due to their small number, no further analyses were done on the OR.

### Nutrient and food intake estimates in under-reporters and acceptable reporters

UR had a lower percentage of energy derived from fat compared to AR (p < 0.01), whereas the percentage of energy from protein was higher for UR than for AR (p = 0.01). No differences in percentage of energy from carbohydrates or added sugar were observed comparing UR and AR (Table 2). UR showed significantly lower absolute intake of vegetables compared to AR (p = 0.03). There were no significant differences between UR and AR in the absolute intakes of berries, chocolate, coffee, fruit, fruit and berry juices, nuts, tea and wine (Table 2). However, significantly lower absolute intakes of grain products, potatoes, meat, egg, dairy products and butter and margarine were observed for UR as compared to AR (under-reported 23, 30, 19, 50, 37 and 69%, respectively, data not shown). When adjusted for EI, differences in food intakes between UR and AR were only observed for butter and fat (p = 0.01, under-reported 38%). Total antioxidant intake was calculated based on total food intake from the FFQ and no significant difference was observed for the total antioxidant intakes of UR and AR (p = 0.14), whereas lower estimates were observed for β-carotene (p = 0.04) and for vitamin C (p = 0.02) in UR as compared to AR (Table 2). With energy-adjustment there were no differences in intakes of antioxidants, β-carotene or vitamin C between UR and AR (antioxidants: p = 0.2; β-carotene: p = 0.6; vitamin C: p = 0.9).

**Table 2:** Percent energy from macronutrients and intakes of nutrients and foods among under-reporters and acceptable reporters

	**UR, n = 19**		**AR, n = 35**	
	
	**Mean**	**SD**	**Mean**	**SD**
	
Percent energy from				
added sugar	4	2.4	5	2.4
carbohydrates	45	5.0	42	5.5
fat	30^a^	6.2	35	5.3
protein	18^b^	2.4	17	2.0

Intakes of	Median	25^th ^, 75^th ^ percentiles	Median	25^th ^, 75^th ^ percentiles
	
antioxidants (mmol/d)	20	12, 25	24	14, 29
vitamin C (mg/d)	118^c^	76, 159	151	114, 198
β-carotene (mcg/d)	2^d^	1, 3	3	2, 4
berries (g/d)	13	1, 29	19	10, 30
chocolate (g/d)	6	1, 13	7	4, 22
coffee (g/d)	442	66, 617	406	36, 679
fruit (g/d)	121	69, 221	113	66, 181
fruit and berry juices (g/d)	42	11, 86	107	21, 185
nuts (g/d)	3	1, 9	6	2, 16
tea (g/d)	80	20, 400	60	20, 220
vegetables (g/d)	212^e^	110, 252	265	199, 414
wine (g/d)	66	12, 119	43	3, 89

Under-reporters, UR; acceptable reporters, AR; standard deviation, SD. ^a ^Different from AR, p = 0.003; ^b ^different from AR, p = 0.012; ^c ^different from AR, p = 0.024; ^d ^different from AR, p = 0.040; ^e ^different from AR, p = 0.027

### Relative validity of food and nutrient intakes

For participants in the WR study, mean age was 48 (95% CI 44, 52) years, mean BMI was 24.8 (95% CI 24.2, 25.4) kg/m^2 ^and 28 were current smokers (15 men and 13 women). Total EI estimates were 9.1 and 9.4 MJ/day, from the FFQ and WR respectively, and did not differ significantly (p = 0.2). However, percent energy from added sugar, total carbohydrates, fat and protein all differed significantly between the FFQ and the WR (Table 3). The absolute intakes of β-carotene and vitamin C were not significantly different between the two methods, whereas the antioxidant intake estimates were higher assessed by the FFQ than by the WR (Table 3). Spearman correlation coefficients for antioxidants, vitamin C and β-carotene did all increase by 0.1 when energy adjusted (data not shown).

**Table 3:** Percent energy from macronutrients and nutrients and food estimates from the FFQ and WR

	FFQ	WR	Ratio FFQ/WR	Correlation^a^
	
	Mean (SD)	Mean (SD)	Mean (SD)	
Percent energy from				
added sugar	5 (3.8)^b^	8 (3.9)	0.76 (0.54)	0.60^c^
carbohydrates	44 (6.1)^b^	42 (6.8)	1.06 (0.16)	0.51^c^
fat	33 (5.3)^b^	36 (5.6)	0.93 (0.17)	0.40^c^
protein	17 (2.4)^b^	16 (2.9)	1.08 (0.17)	0.55^c^
Intakes of	Median (P25, P75)	Median (P25, P75)	Median (P25, P75)	
	
antioxidants (mmol/d)	20 (15, 28)^d^	18 (12, 24)	1.16 (0.94, 1.57)	0.60^g^
vitamin C (mg/d)	136 (102, 178)	128 (88, 179)	1.12 (0.72, 1.82)	0.29^g^
β-carotene (mcg/d)	2402 (1508, 3519)	2079 (1225, 3101)	1.15 (0.70, 1.92)	0.38^g^
berries (g/d)	21 (13, 32)^d^	11 (3, 24)	1.55 (0.83, 3.25)	0.41^g^
chocolate (g/d)	7 (2, 17)	8 (0, 22)	0.63 (0.32, 1.91)	0.58^g^
coffee (g/d)	380 (89, 645)^e^	302 (99, 495)	1.10 (0.81, 1.81)	0.78^g^
fruit (g/d)	151 (80, 228)	127 (70, 248)	1.02 (0.71, 1.67)	0.61^g^
fruit and berry juices (g/d)	60 (19, 150)	73 (0, 178)	0.80 (0.38, 1.51)	0.57^g^
nuts (g/d)	6 (3, 12)	2 (0, 14)	0.65 (0.32, 1.44)	0.40^g^
tea (g/d)	90 (20, 400)^f^	80 (0, 298)	1.05 (0.55, 1.80)	0.74^g^
vegetables (g/d)	243 (166, 322)^d^	162 (118, 222)	1.39 (1.01, 2.03)	0.38^g^
wine (g/d)	45 (14, 106)	48 (0, 114)	0.96 (0.46, 1.49)	0.70^g^

Food frequency questionnaire, FFQ; weighed food record, WR; standard deviation, SD. N = 147. ^a ^all correlations were significant at the 0.01 level; ^b ^different from WR, p < 0.01; ^c ^Pearson correlation; ^d ^different from WR, p < 0.001, ^e ^different from WR, p = 0.003; ^f ^different from WR, p = 0.001, ^g ^Spearman correlation

Estimated intakes of antioxidant-rich foods from the two methods, ratios and correlation coefficients are shown in Table 3. The intakes of berries, coffee and tea from the FFQ were higher than the intakes estimated from the WR (Table 3). No significant differences were observed for the absolute intakes of chocolate, fruit, fruit and berry juices, nuts or wine. Similar results were observed for energy-adjusted intakes (data not shown). Spearman correlation coefficients ranged from 0.40 for nuts to 0.78 for coffee (Table 3).

### Cross-classification of nutrient and food intake

Classification of participants into quartiles of intake showed that approximately 80% of the participants were classified into correct or adjacent quartile when considering percent energy from macro nutrients (Table 4). For antioxidant-rich foods and beverages the fraction of participants classified into correct or adjacent category ranged from 74% for vegetable intake to 96% for coffee and tea intakes. Only 1% of the participants were grossly misclassified, for intakes of chocolate, coffee, fruits, nuts, tea and wine, whereas 6% were grossly misclassified according to berry and vegetable intakes. Energy adjustment of food and nutrient estimates resulted in only small changes in classification (data not shown).

**Table 4:** Cross-classification of subjects according to intake estimates

	Classified correctly	Classified correctly or adjacent	Grossly misclassified by 3 categories
% energy from added sugar	40	81	3
% energy from carbohydrates	39	81	1
% energy from fat	36	78	4
% energy from protein	38	86	2
Intakes of			
antioxidants	48	84	4
vitamin C	33	70	6
β-carotene	36	75	4
berries	37	78	6
chocolate	45	86	1
coffee	59	96	1
fruits	49	84	1
fruit and berry juices	44	86	2
nuts	44	82	1
tea	64	96	1
vegetables	39	74	6
wine	54	93	1

Percent of participants classified correctly, correctly and adjacent and grossly misclassified, respectively, according to percent energy form macro nutrients and intakes of antioxidants and antioxidant-rich foods and beverages, n = 147.

## Discussion

### Evaluation of energy intake

The present evaluation study shows that the FFQ underestimates the EI by 11%. This level of underreporting is comparable to what was found in the validation study of the original NFFQ using doubly labelled water [5], and lower than what has been reported from some earlier European studies [17,18]. A Danish validation study using ActiReg^® ^for EE assessment and a pre-coded food diary for EI assessment showed a similar degree of under-reporting [19]. The proportion of URs in our study (32% of the participants) is slightly higher than reported from the EPIC study [17] but lower than what was reported from the OPEN study [18], and from an earlier national survey in Norway [20]. The tendency to under-report has in some studies been associated with body weight or BMI [21], but it is not confined to obese people and studies have shown that other factors like socio-demographic factors, lifestyle, psychosocial factors, education and characteristics of diet may be related to under-reporting [21-26]. These factors were not assessed in our present study. We found no evidence of association between body weight, smoking, BMI or EE and under-reporting in our present study.

Furthermore, in our study population including both men and women we observed a moderately high correlation coefficient between EE and EI, comparable to the study by Biltoft-Jensen and colleagues using ActiReg^® ^as the reference method [19]. However, there was no correlation between EI and EE for women only. Our data showed that two women grossly over-reported their EI whereas 4 other women underreported their EI by more than 42%. These outliers had a pronounced effect on the Pearson correlation coefficient and we speculate that including a higher number of female participants would have decreased the impact of these extreme reporters [27]. Excluding the 6 extreme reporters resulted in a correlation coefficient between EI and EE for the remaining women of r = 0.3 (data not shown). In future studies employing the FFQ, it might be considered to exclude the 5% highest under- and over reporters when food and nutrient intakes are estimated or subjects are to be ranked according to intake.

The correlations between EI and EE observed for UR and AR separately were similar and high, suggesting that the FFQ has good ability to rank participants according to EI, although the correlation independent of reporting category was hampered by the under-reporting of EI done by the UR. The fraction of participants classified into correct tertiles of EI showed that the FFQ had a satisfactory ability to rank participants in correct and adjacent tertiles, with less than 10% grossly misclassified. However, the FFQ showed a higher frequency of misclassification of EI among women than men.

### Nutrient and food intakes among under-reporters and acceptable reporters

Comparison of EI against EE identifies bias only in reporting of EI. The identified underreporting raises questions as to whether the diet is underreported as a whole, or whether there is selective under-reporting of different foods leading to further bias in the reporting of nutrient intake. Studies among adults suggest that UR report low consumption of all food groups, but that the degree of under-reporting differs between foods perceived as healthy and unhealthy [20,28-32]. In earlier validation studies, UR showed a lower intake of fat and simple carbohydrates as compared with AR [20,28,30,32]. In our present study, UR showed lower percentage of energy from fat but not from added sugars or total carbohydrates, compared to AR. This corresponds to the observed low intakes of energy-dense foods like butter and margarine. The observed difference between UR and AR in intake of vegetables may have consequences for the absolute intakes of vitamin C and β-carotene in UR, which were slightly but significantly lower than in AR. However, energy adjustment of the food intakes showed that the relative intake of antioxidant-rich foods were similar in UR and AR.

### Relative validity of nutrients, food and beverage intakes

The 7-days WR is an open-ended dietary assessment method that captures more details and variation in the diet than the closed method of the FFQ [1]. In our present study the percentage of energy derived from added sugar and fat were lower in the FFQ than in the WR, whereas percentage of energy from total carbohydrates and protein were higher in the FFQ. The average intake of antioxidants was significantly overestimated by the FFQ whereas the correlation was high. The lower correlations observed for vitamin C and β-carotene corresponded to the low correlation for vegetable and berry intake. Consumption of berries is often seasonal and over a year the intake may vary greatly. Although the FFQ included questions concerning berry intake within as well as outside berry season, the intake of berries showed pronounced degrees of over-reporting and misclassification. The individual WR recording periods were distributed from September through March. Not including the summer months, the typical strawberry season in Norway, could be a disadvantage and contribute to reduce the strength of the association between the FFQ and the WR berry intakes. Future dietary assessments using the FFQ should consider this tendency to over-report berry intake and correct for this in the estimates. However, for the other antioxidant-rich foods and beverages; chocolate, coffee, fruit, fruit and berry juices, nuts, tea and wine, the correlations between the two methods were good, especially for coffee, tea and wine, and the fraction of grossly misclassified participants were low. The intake of fruit, fruit juices and vegetables will be further evaluated using plasma and urine biomarkers.

### Strengths and limitations of the evaluation study

Studies of the validity of FFQs are often difficult to carry out due to the difficulties of obtaining a sufficiently large and representative sample of the population to which the FFQ may be applied, and the lack of a 'gold standard' reference method. The participants in our study were recruited from a restricted geographical area of the country and only 11% of those invited responded, suggesting that the study population was biased towards motivated subjects. The age distribution in the present study population was comparable to the distribution of age in this area (Statistics Norway, SSB august 2009, http://www.ssb.no/). There were only small deviations in relation to gender and smoking; our study population had more women (2%) and fewer smokers (3%) than the average for this geographical area of Norway. Moreover, local dietary variations throughout the country may suggest that the inhabitants of the Norwegian capital and surrounding area may not be representative of the general Norwegian population with regard to habitual diet. Sample size calculations were performed based on the ability to detect differences in intake and not based on correlation estimations. Thus, the sample sizes of the genders separately were too small to detect correlations below 0.47.

Prolonged recording like 7-days WR can lead to misreporting of food intakes because the recording itself may be too demanding for the participants [33], resulting in changes in behavior and dietary intake [34]. Moreover, the list of food items in the FFQ may not cover a participant's habitual diet whereas the WR may not capture the habitual intake of foods consumed sporadically because it does not cover the same time period as the FFQ. Misreporting of food intake will promote attenuation of the correlation between the FFQ and the reference method [1]. One of the strengths of our present study is that we used an objective instrument as reference method to measure EE. The ActiReg^® ^system has been validated, demonstrating no significant difference between EE measured with ActiReg^® ^and EE measured with double labeled water or indirect calorimetry [8]. However, ActiReg^® ^shows considerable variation at the individual level. Certain types of arm work, carrying loads and water activities are not well accounted for due to the design of the instrument. Moreover, conclusions about the validity of the FFQ EI estimates can only be drawn if two underlying assumptions hold true: that the participants are in energy balance and that the short term EE measurement represents the habitual and usual EE. In our present study EE was measured using the ActiReg^® ^system during 7 consecutive days. Fluctuations in energy balance during these 7 days were not assessed, thus we can only assume the participants were in energy balance during this period. With respect to the second assumption there was only one 7-days EE measurement period, whereas the FFQ was designed to assess usual dietary intake over the preceding year. The observed discrepancies between EE and EI might therefore partly be explained by the incomplete coverage of the FFQ data by the ActiReg^® ^measurement period.

The implications of the errors in energy, food and nutrient intakes identified in our present study will have attenuating effects on the risk assessments in future epidemiological studies employing the FFQ [1], and may require increased sample size due to reduction in power [35].

## Conclusions

In conclusion, our new FFQ provides a good estimate for the average EI, underestimating the EI by 11% as compared to the EE and by 5% as compared to the WR. The ability of the FFQ to assess average intakes of antioxidant-rich foods and beverages was for most items good. The FFQ's ability to rank participants according to intake of total antioxidants and most of the antioxidant-rich foods was also good.

## List of abbreviations

FFQ: Food Frequency Questionnaire; NFFQ: NORKOST food frequency questionnaire; EE: energy expenditure; EI: energy intake; BMI: body mass index; WR: weighed food record; UR: under-reporters; AR: acceptable reporters; OR: over-reporters; SD: standard deviation.

## Competing interests

RB and CAD are shareholders in Vitas AS. The other authors declare that they have no competing interests.

## Authors' contributions

MHC was responsible for study design, design of the FFQ, recruitment, data collection, statistical analyses and preparation of manuscript. ITLL contributed to study design, data collection, statistical analyses and manuscript revision, AK contributed to study design, recruitment, data collection and manuscript revision, RB was responsible for funding, study design and manuscript revision, CAD contributed to study design and manuscript revision and LFA contributed to study design, design of the FFQ, statistical analyses and manuscript revision. All authors read and approved the final manuscript.
